# *In vitro* evaluation of the effect of C-4 substitution on methylation of 7,8-dihydroxycoumarin: metabolic profile and catalytic kinetics

**DOI:** 10.1098/rsos.171271

**Published:** 2018-01-10

**Authors:** Yang-Liu Xia, Tong-Yi Dou, Yong Liu, Ping Wang, Guang-Bo Ge, Ling Yang

**Affiliations:** 1 Laboratory of Pharmacology & Toxicology, School of Life Science and Medicine, Dalian University of Technology, Panjin 124221, People's Republic of China; 2 Institute of Interdisciplinary Integrative Medicine Research, Shanghai University of Traditional Chinese Medicines, Shanghai 201203, People's Republic of China

**Keywords:** 7, 8-dihydroxycoumarins, human catechol-*O*-methyltransferase, structure–methylation relationship, metabolic stability

## Abstract

Daphnetin (7,8-dihydroxycoumarin (7,8-DHC)) and its C-4 derivatives have multiple pharmacological activities, but the poor metabolic stability of these catechols has severely restricted their application in the clinic. Methylation plays important roles in catechol elimination, although thus far the effects of structural modifications on the metabolic selectivity and the catalytic efficacy of human catechol-*O*-methyltransferase (COMT) remain unclear. This study was aimed at exploring the structure–methylation relationship of daphnetin and its C-4 derivatives, including 4-methyl, 4-phenyl and 4-acetic acid daphnetin. It was achieved by identifying the methylated products generated and by careful characterization of the reaction kinetics. These catechols are selectively metabolized to the corresponding 8-*O*-methyl conjugates, and this regioselective methylation could be elucidated by flexible docking, in which all the 8-OH groups of these catechols are much closer than the 7-OH groups to catalytic residue LYS144 and methyl donor AdoMet. The results of the kinetic analyses revealed that the Cl_int_ values of the compounds could be strongly affected by the C-4 substitutions, which could be partially explained by the electronic effects of the C-4 substituents and the coordination modes of 7,8- dihydroxycoumarins in the active site of COMT. These findings provide helpful guidance for further structural modification of 7,8-DHCs to improve metabolic stability.

## 1. Introduction

Coumarins are widely distributed in food and Chinese medical herbs. With excellent safety, high solubility, good permeability and extensive pharmacological activities, this family has received widespread attention [1]. Daphnetin (7,8-dihydroxycoumarin (7,8-DHC)) is a naturally occurring catechol from the family Thymelaeaceae. Similar to other coumarin derivatives, daphnetin has been reported to have many pharmacological actions including anti-malarial, anti-arthritic, anti-pyretic and anti-cancer properties [2–5]. 4-Methyl-7,8-DHC was found to exhibit important antioxidant strength, a low cytotoxicity, and could decrease reactive oxygen species production in malignant cell lines [6]. Despite the therapeutic benefits, this family suffers from low oral bioavailability due to poor metabolic stability [7,8]. Pharmacokinetic study after administration in rats shows that daphnetin is rapidly eliminated with quite a short half-life (15 min), due to extensive first-pass metabolism in the liver [7,8].

Owing to the existence of phenolic hydroxyl groups, catechol coumarins could be expected substrates of phase II metabolizing enzymes. In general, hydroxycoumarins could be metabolized by glucuronidation catalysed by uridine-5-diphosphate glucuronosyl transferases or sulfation catalysed by sulfotransferases, as is seen for 7-hydroxycoumarin and 4-methylumbelliferone [9,10]. Taking the catechol moiety into consideration, catechol-O-methyltransferase (COMT) may also participate in the metabolic elimination of catechol coumarins *in vivo*, such as fraxetin and daphnetin [11,12]. Recent studies demonstrated that daphnetin can be extensively metabolized in human liver to form six conjugated metabolites [13]. Compared with glucuronidation and sulfonation pathways, the methylation of daphnetin had a much higher clearance rate in human liver S9 fractions and contributed to a large amount (37.3%) of the methyl-derived metabolites in human hepatocytes. Reaction phenotyping studies showed a major role of soluble COMT in daphnetin 8-*O*-methylation. Because of the wide distribution of COMT in human liver, intestine, lung, brain and platelets etc., there is no doubt that methylation is essential in the disposition of these catecholic coumarins [14,15]. Methylation is a crucial pathway for daphnetin clearance and might be involved in pharmacologic actions of daphnetin in humans. However, the structure–methylation relationship of a series of 7,8-DHC analogues remains unclear; understanding of this relationship is necessary for designing structural modifications to improve their metabolic stability.

**Figure 1.**
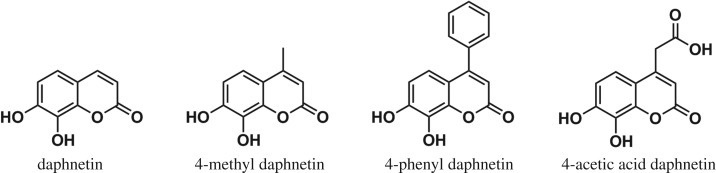
Structures of daphnetin and its C-4 derivatives.

For the present case study, we selected four 7,8-DHC derivatives, daphnetin, 4-methyl daphnetin (4-MDPN), 4-phenyl daphnetin (4-PDPN) and 4-acetic acid daphnetin (4-ADPN) (figure 1), which contain varied groups substituted in C-4 position as model compounds. The effects of altering the substituent within the C-4 position of 7,8-DHC on the catalytic behaviour of COMT were explored. To the best of our knowledge, no other comparative or systematic studies have yet been published to address these aspects of the structure–methylation relationships of 7,8-DHCs.

## 2. Material and methods

### 2.1. Material

Daphnetin, 4-MDPN and 4-PDPN (purity > 98%) were purchased from J&K (Beijing, China). 4-ADPN was synthesized by the author (P.W.) and fully characterized by LC-MS, ^1^H-NMR and^13^C-NMR. Magnesium chloride, dl-dithiothreitol (DTT) and *S*-(5′-adenosyl)-l-methionine (SAM) *p*-toluenesulfonate salt were purchased from Sigma-Aldrich (St. Louis, MO, USA). Pooled human liver cytosol (HLC) from seven male donors (Lot. QROX) was purchased from the Research Institute for Liver Diseases (Shanghai, China). Recombinant S-COMT was expressed and purified by the author (T.-Y.D.). All other reagents were of HPLC grade or of the highest grade commercially available.

## 3. Methods

### 3.1. Methylation assays

Daphnetin and its derivatives at various concentrations were incubated with HLC or recombinant human soluble COMT (S-COMT) in a reaction mixture containing 50 mM Tris–HCl buffer (pH 7.4), 5 mM MgCl_2_ and DTT 4 mM in a final volume of 200 µl. Reaction mixtures were pre-incubated at 37°C for 5 min and initiated by the addition of SAM to a final concentration of 0.4 mM. The samples were incubated for a certain period of time and the reaction was terminated by the addition of 200 µl ice-cold acetonitrile and then centrifuged at 20 000*g*, 4°C, for 20 min. Control incubations were performed either without human liver cytosol, or without SAM or without substrate. The supernatants were subjected to ultra-fast liquid chromatography (UFLC) coupled with a diode array detector (DAD) and an electrospray ionization (ESI) mass spectrometer (MS) analyzer.

### 3.2. Analytical methods

7,8-DHCs and their methylated conjugates were analysed by a UFLC spectrometry system (Shimadzu, Kyoto, Japan), equipped with two LC-20AD pumps, a DGU-20A3 vacuum degasser, a SIL-20ACHT auto-sampler, a CTO-20AC column oven, an SPD-M 20A DAD, a CBM-20A communications bus module, a mass detector (2010EV) with an ESI interface and a computer equipped with UFLC-MS Solution v. 3.41 software. A Shimadzu ODS (75.0 × 2.0 mm, 3 µm) analytical column was used to separate 7,8-DHCs and their methylated conjugates. Column temperature was kept at 40°C. The mobile phase was acetonitrile (A) and 0.2% formic acid (B) at a flow rate of 0.4 ml min^−1^, with a gradient: 0–10.0 min, 90% B–30% B (and 5% for 4-PDPN); 10.0–13.0 min, 5% B; 13.0–16.0 min, balance to 90% B. The maximum absorption wavelengths for daphnetin, 4-MDPN, 4-PDPN and 4-ADPN were 322 nm, 323 nm, 325 nm and 326 nm, respectively. The methylation samples were stable over 72 h at 4°C. 7,8-DHC methylation was quantified by the standard curves of the methylated conjugates at the detector wavelength of 325 nm, which were linear from 0.01 to 10 µM (the correlation coefficient was 0.999). The quantitative method displayed good sensitivity, with the limit of detection of methylated conjugates of 7,8-DHCs below 0.5 ng. The method also displayed good reproducibility, with the intraday and interday variance both less than 3%.

Mass detection was performed on a Shimadzu LC-MS-2010EV instrument with an ESI interface in both positive and negative ion mode from *m/z* 100 to 800. The detector voltage was set at +1.55 kV and −1.55 kV, for positive and negative ion detection, respectively. The curved desolvation line (CDL) temperature and the block heater temperature were set at 250°C, while the CDL voltage was set at 40 V. Other MS detection conditions were as follows: interface voltage, +4.5 kV and −4.0 kV for positive and negative ion detection, respectively; nebulizing gas (N_2_) flow was 1.5 l·min^−1^ and the drying gas (N_2_) pressure was set at 0.06 MPa. Data processing were performed using the LC/MS Solution v. 3.41 software (Shimadzu, Kyoto, Japan).

### 3.3. Biosynthesis of metabolites and their structural identification

The major methylated conjugate of each substrate including daphnetin, 4-MDPN, 4-PDPN and 4-ADPN was biosynthesized using recombinant human S-COMT and purified for structure elucidation and quantitative analysis. In brief, substrate (1 mM) was incubated with recombinant human S-COMT (0.2 mg protein per ml), 0.1 M Tris–HCl (pH 7.4), 10 mM MgCl_2_, 4 mM DTT and 5 mM SAM in 1 ml of final incubation for 4 h at 37°C. This analytical-scale reaction was scaled up to a volume of 48 ml for each substrate. The stock solution of substrate (100 mM) was prepared in DMSO. The concentration of organic solvent in the final incubation was 1%. The reaction was terminated by the addition of ice-cold methanol, and then the vessels were transferred to an ice bath and cooled for 20 min. After the removal of protein by centrifugation at 20 000*g* for 30 min at 4°C, the combined supernatants were loaded on a solid phase extraction (SPE) cartridge (C18PN, 1000 mg, Acchrom Technologies, Beijing, China), which was preconditioned by sequential washing with 6 ml methanol and 6 ml Millipore water. After sample loading, the SPE cartridge was sequentially eluted with 12 ml Millipore water, 12 ml methanol and 12 ml methanol containing 5% formic acid. The entire process was monitored by UFLC-UV, and the methylated conjugate was collected in methanol containing 5% formic acid. After vacuum evaporation, the methylated conjugate of each 7,8-DHC was obtained as a powder and the purity was greater than 96%. The structure of each methylated conjugate was characterized by the NMR technique including ^1^H-NMR and ^13^C-NMR. All experiments were carried out on a Bruker Avance-400 NMR spectrometer (Bruker, Switzerland). Each methylated conjugate was stored at −80°C before dissolving in DMSO-d_6_ (Euriso-Top, Saint-Aubin, France) for NMR analysis. Chemical shifts were given on *δ* scale and referenced to tetramethylsilane at 0 ppm for ^1^H-NMR (400 MHz) and ^13^C-NMR (150 MHz).

### 3.4. Kinetic analysis

For estimating kinetic parameters, 7,8-DHCs (0.1–40 µM) were incubated with pooled HLC and S-COMT. 7,8-DHCs (0.1–40 µM) were incubated with HLC or S-COMT for 5 min, using a microsomal protein concentration of 0.5 mg ml^−1^ or 0.002 mg ml^−1^ for HLC and S-COMT, respectively. Kinetic constants for 7,8-DHCs methylation by HLC and S-COMT were obtained by fitting the Michaelis–Menten kinetics equation (3.1) to experimental data.

For the Michaelis–Menten kinetic model,

3.1 $$v=\frac{V_{\max}\times [S]}{K_{m}+[S]},$$

where *v* is the rate of reaction, [*S*] is the substrate concentration, *V*_max_ is the maximum velocity estimate and *K*_m_ is the apparent affinity constant.

All incubations were performed in duplicate. Kinetic constants were obtained using GraphPad Prism 6 (GraphPad Software, Inc. La Jolla, CA) software.

### 3.5. Docking simulation of 7,8-dihydroxycoumarins into the reported structure of COMT

Flexible docking was performed using Discovery Studio (BIOVIA Discovery Studio 2016, Dassault Systèmes, San Diego, USA) [16]. The protein structures of human COMT were taken from the Protein Data Bank [17,18]. Tasks including inserting missing atoms in incomplete residues, modelling missing loop regions, deleting alternate conformations (disorder), standardizing atom names and protonating titratable residues using predicted p*K*_s_ were performed. Meanwhile, the four compounds were prepared by removing duplicates, enumerating isomers and tautomers, ionization and generating three-dimensional conformations before being used for the docking process. In the ionization process, the four compounds were prepared for treatment at pH 7.5. Herein, the anionic form of 4-ADPN was assumed in the following docking simulation. The CHARMM 40.1 force field was used to represent both the protein and ligand structures. Flexible docking, a fully automated molecular mechanics based induced-fit protein--ligand docking method, includes the following steps: (i) calculates receptor conformations using ChiFlex (a modified version of the ChiRotor sampling algorithm); (ii) creates ligand conformations; (iii) performs ligand docking into each active protein conformation site using LibDock; (iv) clustering to remove similar ligand poses regardless of the protein conformation; (v) refines selected protein side-chains in the presence of the rigid ligand using ChiRotor; and (vi) performs a final ligand refinement using CDOCKER [18,19]. Here in this work, residues included in the active site AC4 of 3BWY (the Protein Data Bank (PDB) code for the structure of human COMT), TRP38, MET40, ASP141, TRP143, LYS144, ASP169, ASN170, LEU198, GLU199, Mg^2+^ and AdoMet, are selected to undergo conformation changes during docking. The distances between hydroxyl groups of 7,8-DHCs and the LYS144 residue, AdoMet and Mg^2+^ were measured.

### 3.6. Molecular calculations

The p*K*_a_ values of 8-hydroxyl groups of 7,8-DHCs were calculated using p*K*_a_ estimation software (MarvinBeans p*K*_a_ estimation plug-in, v. 15.6.29, ChemAxon, Budapest, Hungary).

## 4. Results

### 4.1. Methylation profiles and metabolite characterization

**Figure 2.**
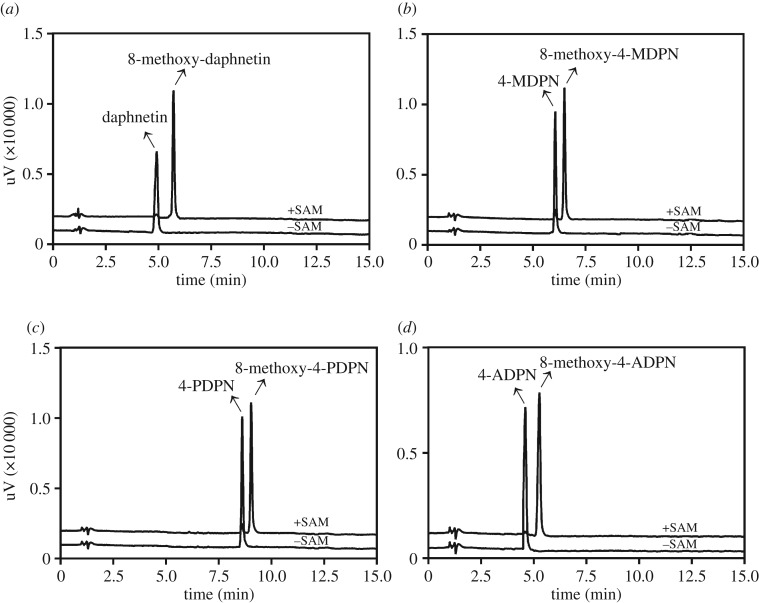
Chromatograms of incubation systems with (*a*) daphnetin, (*b*) 4-MDPN, (*c*) 4-PDPN and (*d*) 4-ADPN. Each substrate was incubated in human liver cytosol with SAM (+SAM) and without (−SAM) for 60 min. The diode-array detector was set at a wavelength of 325 nm.

One product peak was eluted when each 7,8-DHC (10 µM) was incubated with HLC (0.5 mg ml^−1^) in the presence of SAM (figure 2). These peaks were not detected in the control samples without SAM, 7,8-DHCs or HLC. These metabolites were identified by LC-ESI-MS to show identical product ions at *m/z* 191, 205, 265 and 249 in negative-ion mode for daphnetin, 4-MDPN, 4-PDPN and 4-ADPN (increased *m/z* of 14 compared with their corresponding substrates), respectively, indicating that these metabolites were mono-methyl conjugates. Each of the mono-methyl conjugates was biosynthesized using S-COMT as the enzyme source, and each of the 7-*O*-methylated products of daphnetin, 4-MDPN, 4-PDPN and 4-ADPN was also chemically synthesized and fully characterized by ^1^H- and ^13^C-NMR. By comparison of the ^1^H-NMR spectra of the chemically and bio-synthesized mono-methyl products, C-8 hydroxyl proton signal was observed between 9.38 and 9.55 ppm for the former, while C-7 hydroxyl proton signal appeared between 10.33 and 10.49 ppm for the latter. Additionally, the chemically synthesized 7-*O*-methylated products displayed weak fluorescence emission, while the biotransformed conjugates exhibited extremely strong fluorescence emission in TLC under 365 nm. With the help of 7-*O*-methylated standards, the mono-methyl conjugates formed in the COMT-mediated methylation system could be confirmed to be 8-*O*-methylated products. The ^1^H-NMR and ^13^C-NMR spectra of both the 7-*O*- and 8-*O*-methylated conjugates are displayed in the electronic supplementary material, figures S1–S8. The chemical shifts of ^1^H-NMR and ^13^C-NMR of the 7-*O*- and 8-*O*-methylated conjugates are also provided (electronic supplementary material).

### 4.2. Kinetic analysis

**Figure 3.**
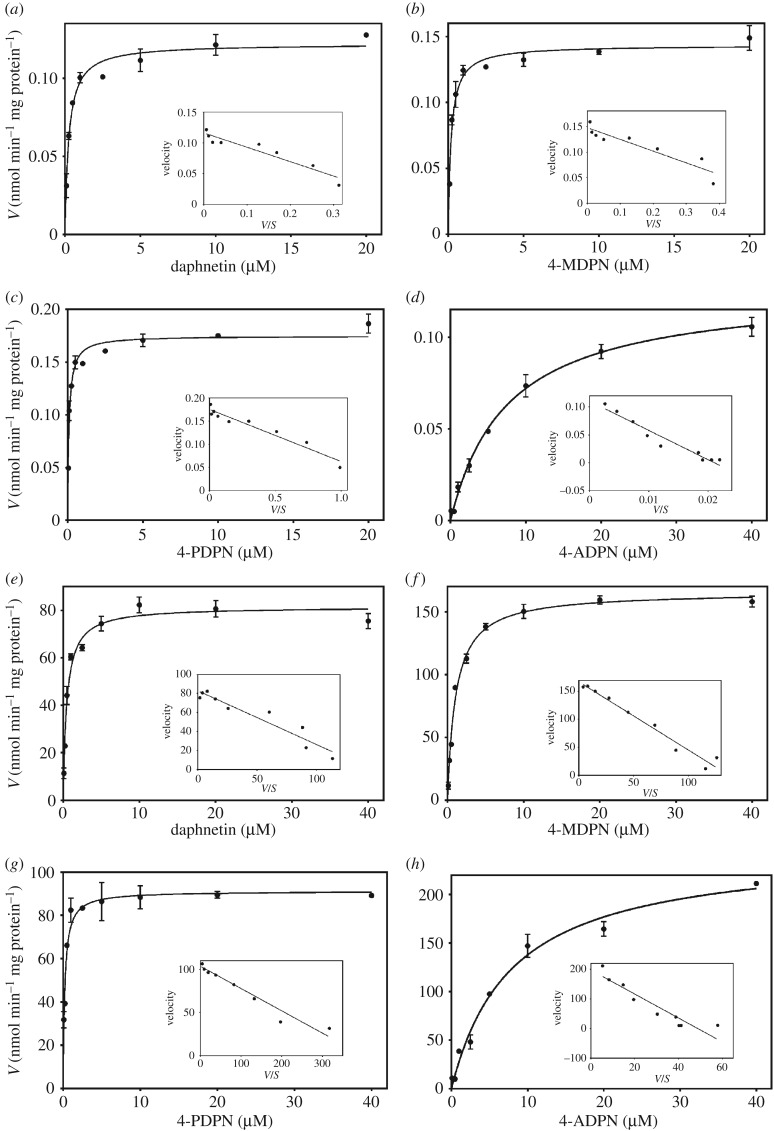
Methylation kinetics of daphnetin and its C-4 derivatives in (*a*–*d*) HLC and (*e*–*h*) recombinant S-COMT. Data were expressed as mean ± s.d.

The kinetic parameters including *K*_m_, *V*_max_ and the intrinsic clearance (*V*_max_/*K*_m_) for methylations of 7,8-DHCs in HLC or S-COMT were determined and listed in table 1. *K*_m_ and *V*_max_ values are expressed as best-fit values ± s.e. The substrate concentrations for the kinetic studies were in the range 0.1–40 µM. Over the whole concentration range tested, methylations of 7,8-DHCs in HLC as well as S-COMT followed Michaelis–Menten kinetics, as evidenced by the Eadie–Hofstee plots (figure 3). Notably, the *K*_m_ value for the methylation of each 7,8-DHC in HLC was close to the *K*_m_ value in S-COMT, implying that *O*-methylation occurring in the cytosol is dominantly catalysed by S-COMT. However, the reactivity (*V*_max_) in 7,8-DHC methylation in S-COMT demonstrated values more than three orders of magnitude higher than those in HLC, which may be due to the high purity of the recombinant S-COMT. Moreover, the *V*_max_ values for the methylations of 7,8-DHCs did not differ too much from each other, but the *K*_m_ values for 7,8-DHCs methylation were altered significantly. Consequently, the Cl_int_ values for the methylation of 7,8-DHCs varied greatly among the four compounds, with the range of 0.017–1.91 µl min^−1^ mg^−1^ in HLC and 31.1–424.0 µl min^−1^ mg^−1^ in S-COMT. The order of Cl_int_ values for 7,8-DHC methylation was 4-PDPN > 4-MDPN ≈ daphnetin > 4-ADPN.

**Table 1.** Kinetics of daphnetin, 4-MDPN, 4-PDPN and 4-ADPN in HLC and S-COMT.

	*K*_m_ (μM)		*V*_max_ (nmol min^−1 ^mg^−1^)		Cl_int_ (μl min^−1^ mg^−1^)	
compound	HLC	S-COMT	HLC	S-COMT	HLC	S-COMT
daphnetin	0.21 ± 0.036	0.48 ± 0.071	0.11 ± 0.0034	81.4 ± 2.4	0.52	169.5
4-MDPN	0.20 ± 0.029	1.07 ± 0.10	0.14 ± 0.0039	165.7 ± 3.5	0.70	154.8
4-PDPN	0.089 ± 0.015	0.22 ± 0.034	0.17 ± 0.0047	93.3 ± 2.4	1.91	424.0
4-ADPN	7.52 ± 0.64	7.93 ± 1.31	0.13 ± 0.0038	247.0 ± 14.6	0.017	31.1

### 4.3. Docking studies of the mode of binding of 7,8-dihydroxycoumarins with human COMT

**Figure 4.**
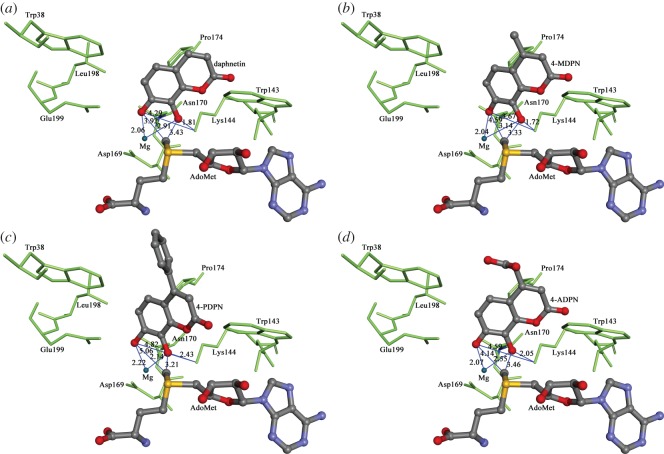
Docking simulation of (*a*) daphnetin, (*b*) 4-MDPN, (*c*) 4-PDPN, (*d*) 4-ADPN into human S-COMT. Distance values are in the unit Å.

To investigate the mechanism of selective 8-*O*-methylation of 7,8-DHCs, flexible docking was performed. The results of CDOCKER indicated that 7,8-DHCs can be well docked into the substrate binding area of human COMT (coded as 3BWY), while the coumarin ring of 7,8-DHCs occupied a hydrophobic pocket located at the catalytic domain (figure 4). In the best docking conformations based on the --CDOCKER_ENERGY, the distances between the C-7 and C-8 phenol group and the amino group of LYS144 were 4.29 Å and 1.81 Å for daphnetin, 3.67 Å and 1.72 Å for 4-MDPN, 4.82 Å and 2.43 Å for 4-PDPN, 4.59 Å and 2.05 Å for 4-ADPN. The corresponding distances between the C-7 and C-8 phenol group and methyl of AdoMet were 3.92 Å and 3.43 Å for daphnetin, 4.59 Å and 3.33 Å for 4-MDPN, 5.06 Å and 3.21 Å for 4-PDPN, 4.14 Å and 3.46 Å for 4-ADPN. The distances between the 7-*O* and 8-*O* atoms and Mg^2+^ were 2.06 Å and 2.91 Å for daphnetin, 2.04 Å and 3.14 Å for 4-MDPN, 2.22 Å and 2.14 Å for 4-PDPN, 2.07 Å and 2.55 Å for 4-ADPN.

### 4.4. Molecular calculations

The p*K*_a_ values of 8-hydroxyl groups were 12.79, 12.77, 12.71 and 12.77 for daphnetin, 4-MDPN, 4-PDPN and 4-ADPN, respectively.

## 5. Discussion

In this study, the interactions of COMT with four 7,8-DHCs, which have varying substituents at the C-4 position, were compared. The four compounds are efficiently metabolized by COMT *in vitro*, each producing different levels of mono-*O*-methyl-ether conjugates. The regioselectivity of *O*-methylation is shown in 7,8-DHCs with different substitution patterns in the C-4 position. We have studied the molecular interactions of the four molecules within the catalytic site to investigate the susceptibility of each of the hydroxyl groups to undergo *O*-methylation by means of docking simulations. Finally, the kinetic parameters of COMT in HLC and S-COMT are disclosed for 7,8-DHCs, and the structure-activity relationship of this type of compound is analysed and discussed.

Our results demonstrated that all of the tested 7,8-DHCs including daphnetin, 4-MDPN, 4-PDPN and 4-ADPN were COMT substrates in human liver cytosol. The methylated metabolite was identified as 8-*O*-methylated 7,8-DHC by NMR, demonstrating that 7,8-DHC is a rare substrate of human COMT favouring a regioselective methylation to form a mono-methylated product [20–22]. As a plausible interpretation of the observed regioselectivities, dominant conformations of the four compounds docked with COMT (based on --CDOCKER_ENERGY) in figure 4 show that for all the 7,8-DHCs the 8-OH group is much closer than the 7-OH group to the catalytic residue LYS144 (approx. 2.0 Å versus approx. 4.2 Å) and the methyl donor AdoMet (approx. 3.4 Å versus approx. 4.3 Å), indicating dominant preference of methylation at the 8-OH rather than the 7-OH group. However, there should be further studies to identify the major structural and chemical factors that determine the preferred methylation sites of 7,8-DHCs.

All of the four 7,8-DHCs could be metabolized by COMT to varying degrees, and more than 10-fold difference in Cl_int_ value between 4-ADPN and 4-PDPN could be observed both in human liver cytosol and in recombinant S-COMT. Considering the catalytic mechanism, COMT-mediated *O*-methylation is reported to be a two-step S_N_2 displacement reaction, which begins with proton transfer from the pre-methylated catechol-OH group to the amino group of Lys144, and then the ionized catechol-*O*- attacks the methyl group of AdoMet to yield the methylation product [14]. Consequently, a decrease in p*K*_a_ value of pre-methylated catechol-OH group would facilitate the first step [22]. In this case, the p*K*_a_ values of the 7- and 8-hydroxyl groups of 7,8-DHCs were determined using p*K*_a_ estimation software, and a relatively lower p*K*_a_ value of 8-hydroxyl of 4-PDPN may partially explain the higher catalytic efficiency of this compound compared with the other three. It could be assumed that the C-4 substitutes may influence the electronic cloud density of the 8-OH group of 7,8-DHCs and further impact the deprotonation process.

Moreover, from the perspective of the enzymatic environment, a slightly more tight coordination of the 8-*O* atom of 4-PDPN to the metal centre, indicated by the shorter distance between the 8-*O* atom of 4-PDPN and Mg^2+^ compared with the other three compounds, may also account for the relatively higher Cl_int_ of 4-PDPN [21]. A more favourable interaction between 4-PDPN and the active site of human COMT could also be observed in our flexible docking simulation. For 4-PDPN, 14 of the 111 docked poses could form the desired octahedral coordination required for the methyl-transfer reaction to occur with the oxygen atoms in the catecholic hydroxyl group coordinating to the Mg^2+^ rigidly with average distances of 2.10–2.30 Å. However, the amount of octahedral coordination formed in the case of the other three compounds is much less. For 4-ADPN, only one out of 57 docked poses are ligated to the Mg^2+^ with average distances of 2.00–2.60 Å. It seems that an orientation effect provided by the C-4 phenyl group which is beneficial to the binding process of 7,8-DHC towards COMT and a relatively high efficiency of ‘correct' binding would be beneficial to the subsequent methyl-transfer step. Hence, the catalytic efficiency is the comprehensive result jointly induced by both the physiochemical properties of the substrate and the interactions between substrate and enzyme. Further work should be conducted to better understand the mechanism accounting for the difference among 7,8-DHCs concerning their COMT-mediated catalysis.

The level of COMT activity in human tissues has been used as a biochemical index to assess individual differences in normal and in pathological conditions, and the availability of a sensitive assay could be useful in the development of new COMT inhibitors [23,24]. However, the applicability of currently available substrates for the measurement of COMT activity in human tissues could be relatively limited owing to the atypical kinetics and time-consuming chromatographic separation of the two methylated products [25,26]. Taken together, the formation of a mono-methylated product and the Michaelis–Menten kinetics of methylation implies that 7,8-DHC derivatives can serve as practical probes for the assessment of COMT activity in complex biological samples *in vitro*. In fact, 4-MDPN has been developed to be a probe for sensing the real activities of COMT in cell and tissue preparations, and this study could provide useful information on the design of COMT probes with high affinity, good reactivity and ideal kinetic behaviours for sensitive quantitative determination of COMT activity [27].

It has been reported that the dihydroxyl moiety of 7,8-DHCs is essential to their biological activities, such as anti-oxidant and anti-cancer activities [28–32]. Accordingly, the retention of catechol phenols is crucial for designing better daphnetin derivatives as therapeutic agents. During the past half century, many coumarin derivatives including a range of C-4 derivatives were synthesized and reported, as these derivatives can be readily synthesized and various substitutes can be easily introduced to the C-4 position by total synthesis or semi-synthesis [33]. Therefore, C-4 modification, such as C-4 acetic acid substitution, is a potential strategy to balance the activity and the metabolic stability, as a relatively low intrinsic clearance was observed for 4-ADPN.

In this work, we carried out the methylation of 7,8-DHCs by COMT using human liver cytosol and recombinant S-COMT. Only 8-*O*-methylated 7,8-DHCs were detected under the experimental conditions applied. A computing model of the enzyme was constructed, and the docking of substrate molecules to the enzyme was performed to verify this regioselective methylation. All of the four 7,8-DHCs were relatively good COMT substrates, while 4-PDPN showed the lowest *K*_m_ value. These findings are in agreement with the docking results using a human S-COMT crystal structure as template. In fact, this constitutes the first report of the systemic structure activity study between COMT and 7,8-DHCs. The results will give us more information about the metabolism of 7,8-DHCs in humans and enhance the knowledge of regioselectivity of methylation by human COMT, which would be very helpful to guide the further structural modification of 7,8-DHCs with improved metabolic stability.

## 6. Conclusion

Our findings demonstrated that the C-8 hydroxyl group is the only metabolic site of 7,8-DHCs metabolized by human COMT, while the C-7 hydroxyl group could hardly be methylated. Furthermore, we also found that the electronic effects of the C-4 substituents on 7,8-DHCs and the coordination modes of 7,8-DHCs in the active site of COMT could strongly affect the catalytic efficiency. All of these findings are very helpful for guiding the further structural modification of 7,8-DHCs with improved metabolic stability.

## Supplementary Material

NMR data of methylated products

## Data accessibility

The dataset supporting this article has been uploaded as electronic supplementary material.
