# Supplementary material for: In vitro evaluation of the effect of C-4 substitution on methylation of 7,8-dihydroxycoumarin: metabolic profile and catalytic kinetics
Source: R Soc Open Sci. 2018 Jan 10;5(1):171271. doi: 10.1098/rsos.171271 (PMC5792912; doi:10.1098/rsos.171271)
Supplement: NMR data of methylated products [file rsos171271supp1.doc]

# (Supporting Information)

# In vitro evaluation of the effect of C-4 substitution on methylation of 7,8-dihydroxycoumarin: metabolic profile and catalytic kinetics

Yang-Liu Xia‡^a^, Tong-Yi Dou‡^a^, Yong Liu^a^, Ping Wang^b^*, Guang-Bo Ge^b^*, Ling Yang^b^

^a^ School of Life Science and Medicine, Dalian University of Technology, Panjin, China,

^b^ Institute of Interdisciplinary Integrative Medicine Research, Shanghai University of Traditional Chinese Medicine, Shanghai, China,

^*^Corresponding author: s200541025@126.com; gegb@dicp.ac.cn;

‡These authors contributed equally to this work.

# Table of Contents

| Description | Page |
| --- | --- |
| Chemical shift of ^1^H-NMR and ^13^C-NMR of methylated products | S2 |
| Fig. S1. ^1^H NMR and ^13^C NMR spectra for compound 8-methoxy daphnetin | S5 |
| Fig. S2. ^1^H NMR and ^13^C NMR spectra for compound 8-methoxy-4-MDPN | S6 |
| Fig. S3. ^1^H NMR and ^13^C NMR spectra for compound 8-methoxy-4-PDPN | S7 |
| Fig. S4. ^1^H NMR and ^13^C NMR spectra for compound 8-methoxy-4-ADPN | S8 |
| Fig. S5. ^1^H NMR and ^13^C NMR spectra for compound 7-methoxy daphnetin | S9 |
| Fig. S6. ^1^H NMR and ^13^C NMR spectra for compound 7-methoxy-4-MDPN | S10 |
| Fig. S7. ^1^H NMR and ^13^C NMR spectra for compound 7-methoxy-4-PDPN | S11 |
| Fig. S8. ^1^H NMR and ^13^C NMR spectra for compound 7-methoxy-4-ADPN | S12 |

#

S1. Chemical shift of ^1^H-NMR and ^13^C-NMR of methylated products

**General Experimental**

The ^1^H NMR and ^13^C NMR spectra were recorded in dimethyl sulfoxide-*d_6_* (DMSO-*d*_6_) using a Bruker ARX 400 spectrometer (400 MHz for ^1^H NMR, 101 MHz for ^13^C NMR), and chemical shifts were expressed as ppm against tetramethylsilane (TMS) as an internal reference. All reagents used in the synthesis were obtained commercially and used without further purification. The reactions were monitored by thin layer chromatography (TLC) on glass-packed precoated silica gel GF_254_ plates and visualized in an iodine chamber or with a UV lamp. Flash column chromatography was performed using silica gel (200~300 mesh) purchased from Qingdao Haiyang Chemical Co. Ltd.

*8-methoxy daphnetin*. ^1^H NMR, δ: 3.82 (s, 3H, OCH_3_), 6.22 (d, *J* = 9.2 Hz, 1H, COCH=C), 6.85 (d, 1H, *J* = 8.4 Hz, Ar-H), 7.26 (d, 1H, *J* = 8.4 Hz, Ar-H), 7.92 (d, *J* = 9.2 Hz, 1H, C=CH), 10.38(s, 1H, Ar-OH). ^13^C NMR, δ: 160.5, 154.3, 148.6, 145.3, 134.6, 124.1, 113.9, 112.6, 111.9, 61.0. M = 192, found 190.9 [M-H]^-^, found 192.9 [M+H]^+^ . IR (KBr), *ṽ*/cm^–1^: 3327, 3080, 2942, 2836, 1570, 1505, 1435, 1343, 1244, 1067.

*8-methoxy-4-MDPN*. ^1^H NMR, δ: 2.36 (s, 3H, CH_3_), 3.82 (s, 3H, OCH_3_), 6.16 (s, 1H, COCH=C), 6.88 (d, *J* = 8 Hz, 1H, Ar-H), 7.35 (d, *J* = 8 Hz, 1H, Ar-H), 10.33 (s, 1H, OH). ^13^C NMR, δ: 160.5, 154.1, 151.1, 142.9, 133.8, 115.6, 114.6, 111.8, 108.6, 56.7, 18.7. M = 206, found 205.0 [M-H]^-^, found 207.0 [M+H]^+^. IR (KBr), *ṽ*/cm^–1^: 3210, 2938, 2839, 1709, 1575, 1513, 1457, 1437, 1369, 1330.

*8-methoxy-4-PDPN*. ^1^H NMR, δ: 3.87 (s, 3H, OCH_3_), 6.18 (s, 1H, COCH=C), 6.86 (d, *J* = 9 Hz, 1H, Ar-H), 7.02 (d, *J* = 9 Hz, 1H, Ar-H) , 7.50-7.57(m, 5H, Ar-H) , 10.49 (s, br, 1H, Ar-OH). ^13^C NMR, δ: 160.2, 156.2, 154.4, 148.8, 135.7, 135.1, 130.0, 129.3, 128.9, 122.5, 113.7, 112.1, 110.9, 61.1. M = 268, found 266.9 [M-H]^-^, found 268.9 [M+H]^+^. IR (KBr), *ṽ*/cm^–1^: 3552, 3447, 3362, 2968, 2840, 1663, 1613, 1574, 1511, 1457, 1444, 1385.

*8-methoxy-4-ADPN*. ^1^H NMR, δ: 3.82 (s, 2H, CH_2_), 3.83 (s, 3H, OCH_3_), 6.26 (s, 1H, COCH=C), 7.89 (d, *J* = 8.8 Hz, 1H, Ar-H), 7.29 (d, *J* = 8.8 Hz, 1H, Ar-H), 10.42 (s, 1H, OH), 12.85 (s, 1H, COOH); ^13^C NMR, δ: 171.1, 160.3, 154.1, 150.9, 148.3, 134.8, 121.0, 113.6, 112.7, 112.6, 61.1, 37.8. M = 250.2, found 249.0 [M-H]^-^.

*7-methoxy daphnetin.* To the solution of dihydroxycoumarin (500 mg, 2.81 mmol) in N,N-dimethylformamide (DMF) 5mL, Na_2_CO_3_ (893 mg, 8.42 mmol) was added at 15 ˚C and stirred for 0.5 h. iodomethane (1.2 g, 525 μL, 8.43 mmol) was added dropwise to the reaction mixture at 15 ˚C, maintaining the temperature at 20 ˚C for 4 h. The reaction mixture was poured into water-ice and acidified with 2 mol/L hydrochloric acid. The reaction mixture was extracted with ethyl acetate (50 mL x 3). The combined organic layer was washed with water and brine, dried over anhydrous sodium sulfate, and evaporated in vacuo. The residue was purified by column chromatography using Silica gel with petroleum ether-dichloromethane-acetone as mobile phase to obtain 7-methoxy daphnetin as white power in 25% yield. ^1^H NMR, δ: 3.88 (s, 3H, OCH_3_), 6.25 (d, *J* = 9.6 Hz, 1H, COCH=C), 7.02 (d, 1H, *J* = 8.8 Hz, Ar-H), 7.15 (d, 1H, *J* = 8.8 Hz, Ar-H), 7.93 (d, *J* = 9.6 Hz, 1H, C=CH), 9.45 (s, 1H, Ar-OH). ^13^C NMR, δ: 160.7, 151.3, 145.3, 143.2, 133.8, 118.9, 113.8, 113.0, 109.0, 56.8. M = 192, found 192.9 [M+H]^+^. IR (KBr), *ṽ*/cm^–1^: 3360, 3059, 2976, 2951, 2848, 1702, 1621, 1567, 1504, 1482.

*7-methoxy-4-MDPN.* To a mixture of 3-methoxybenzene-1,2-diol (0.5 g, 3.5 mmol) and ethyl acetoacetate (0.95 ml, 7.0 mmol) was added drop-wise perchloric acid (3.0 ml) at room temperature and stirred for 6 h. After completion of the reaction as indicated by TLC, the reaction mixture was poured slowly into a mixture of ice-water (50 ml) with stirring. The resultant suspension was filtered and the collected solid was washed with water and dried, then the crude compound was recrystallized from methanol to afford 7-methoxy-4-MDPN as light white solid. ^1^H NMR, δ: 2.38 (s, 3H, CH_3_), 3.89 (s, 3H, OCH_3_), 6.20 (s, 1H, COCH=C), 7.03 (d, *J* = 8 Hz, 1H, Ar-H), 7.21 (d, *J* = 8 Hz, 1H, Ar-H), 9.38 (s, 1H, OH). ^13^C NMR, δ: 160.4, 154.3, 154.1, 148.1, 134.7, 120.9, 113.5, 113.4, 110.8, 61.1, 18.7. M = 206, found 205.1 [M-H]^-^, found 206.9 [M+H]^+^. IR (KBr), *ṽ*/cm^–1^: 3552, 3447, 3362, 2968, 2840, 1663, 1613, 1574, 1511, 1457, 1444, 1385.

*7-methoxy-4-PDPN*. To the solution of 4-phenyl-7,8-dihydroxycoumarin (500 mg, 1.97 mmol) in N,N-dimethylformamide (DMF) 5mL, Na_2_CO_3_ (626 mg, 5.90 mmol) was added at 15 ˚C and stirred for 0.5 h. iodomethane (839 mg, 368 μL, 5.91 mmol) was added dropwise to the reaction mixture at 15 ˚C, maintaining the temperature at 20 ˚C for 4 h. The reaction mixture was poured into water-ice and acidified with 2 mol/L hydrochloric acid. The reaction mixture was extracted with ethyl acetate (50 mL x 3). The combined organic layer was washed with water and brine, dried over anhydrous sodium sulfate, and evaporated in vacuo. The residue was purified by column chromatography using Silica gel with petroleum ether-dichloromethane-acetone as mobile phase to obtain 7-methoxy-4-PDPN as white power in 20% yield. ^1^H NMR, δ: 3.87 (s, 3H, OCH_3_), 6.194 (s, 1H, COCH=C), 6.85 (d, *J* = 9 Hz, 1H, Ar-H), 7.00 (d, *J* = 9 Hz, 1H, Ar-H), 7.49-7.56 (m, 5H, Ar-H), 9.55 (s, br, 1H, Ar-OH). ^13^C NMR, δ: 160.4, 156.0, 151.2, 143.4, 135.7, 134.3, 130.0, 129.2, 128.9, 117.4, 113.3, 111.9, 108.7, 56.7, 49.1. M = 268, found 268.9 [M+H]^+^. IR (KBr), *ṽ*/cm^–1^: 3347, 3000, 2975, 2938, 2839, 1619, 1563, 1509, 1460, 1437, 1380, 1297.

*7-methoxy-4-ADPN.* To a mixture of 3-methoxybenzene-1,2-diol (0.5 g, 3.5 mmol) and 1,3-acetonedicarboxylic acid (1.03g, 7.0 mmol) was added drop-wise perchloric acid (5.0 ml) at room temperature and heated to 50 ^o^C for 6 h. After completion of the reaction as indicated by TLC, a mixture of ice-water (10 ml) was slowly added into the reaction mixture with stirring. The resultant suspension was filtered and the collected solid was washed with water and dried, then the crude compound was recrystallized from acetonitrile to afford 7-methoxy-4-ADPN as light white solid. ^1^H NMR, δ: 3.85 (s, 2H, CH_2_), 3.89 (s, 3H, OCH_3_), 6.30 (s, 1H, COCH=C), 7.05 (d, *J* = 9.2 Hz, 1H, Ar-H), 7.21 (d, *J* = 8.8 Hz, 1H, Ar-H), 9.47 (s, 1H, OH), 12.79 (s, 1H, COOH); ^13^C NMR, δ: 171.2, 160.5, 151.1, 150.7, 143.0, 134.0, 115.7, 113.9, 113.6, 108.8, 56.7, 37.8. M = 250.2, found 249.0 [M-H]^-^

^1^H NMR (400 MHz, DMSO-*d*_6_)


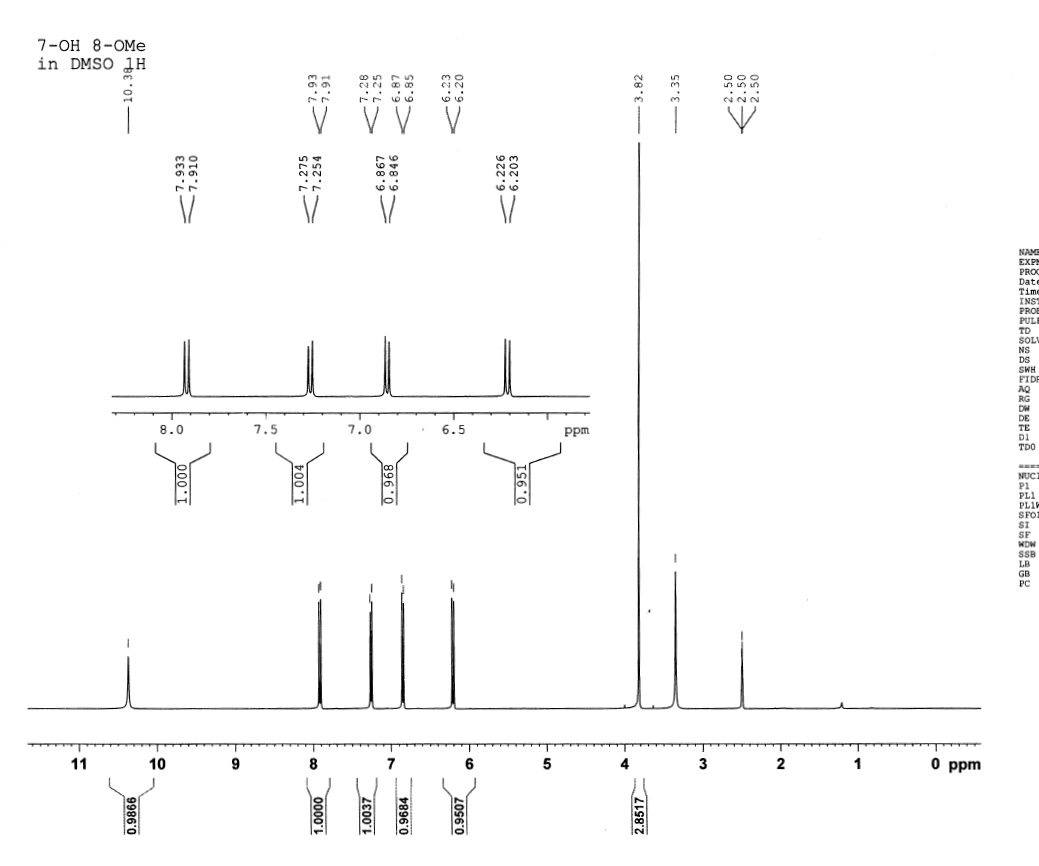


# ^13^C NMR (100 MHz, DMSO-*d*_6_)


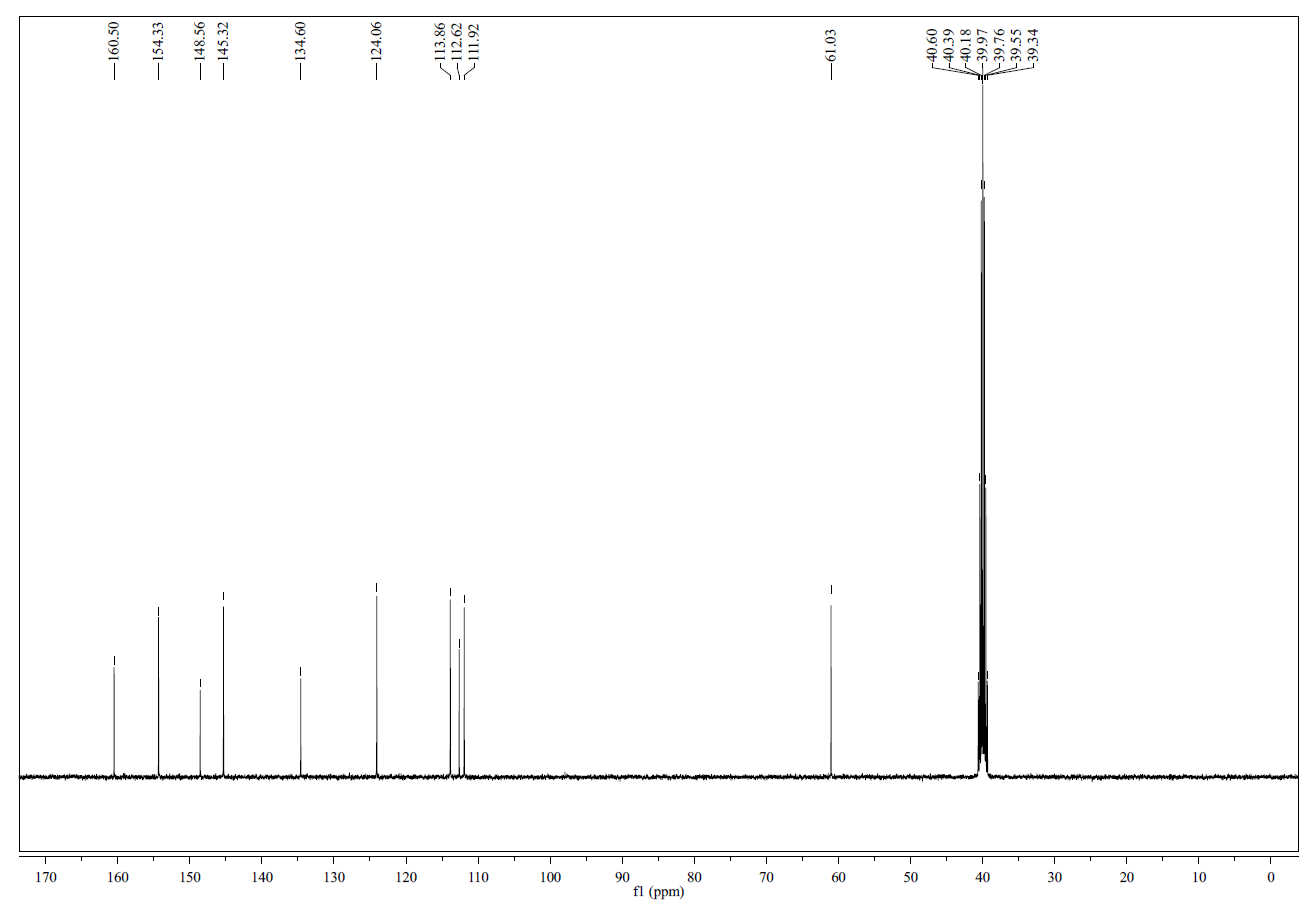


# Fig. S1 ^1^H NMR and ^13^C NMR spectra for 8-methoxy daphnetin

^1^H NMR (400 MHz, DMSO-*d*_6_)


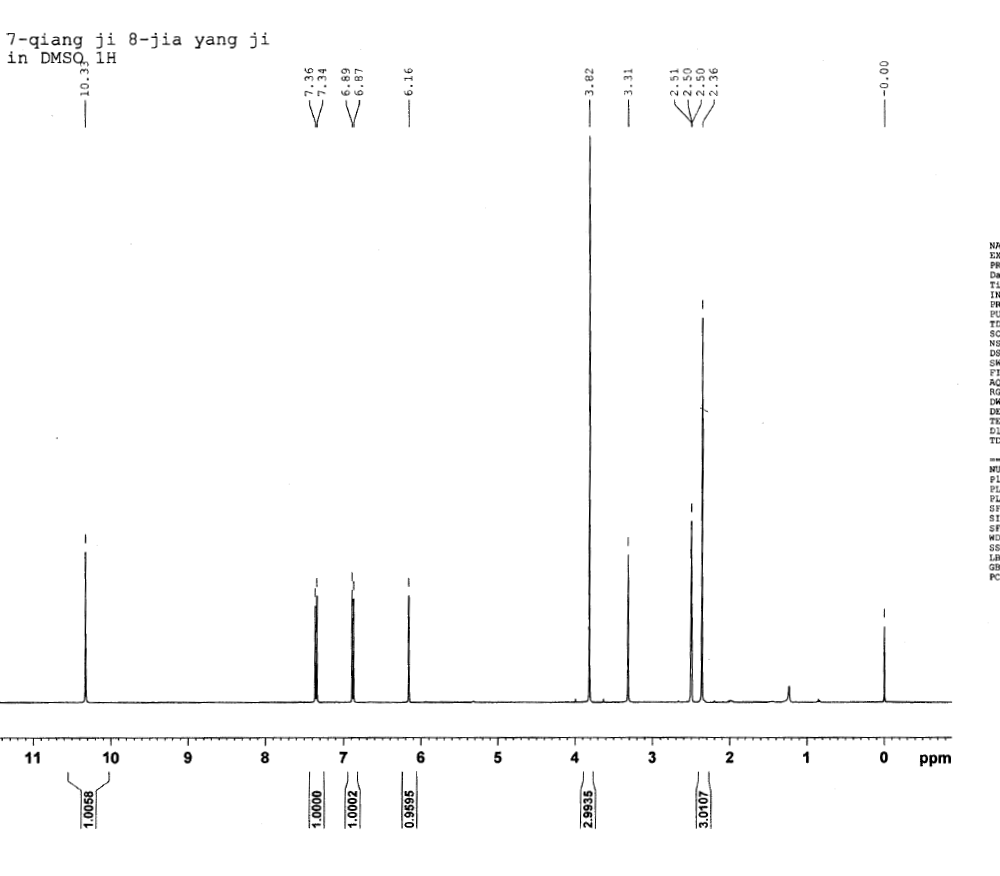


# ^13^C NMR (100 MHz, DMSO-*d*_6_)


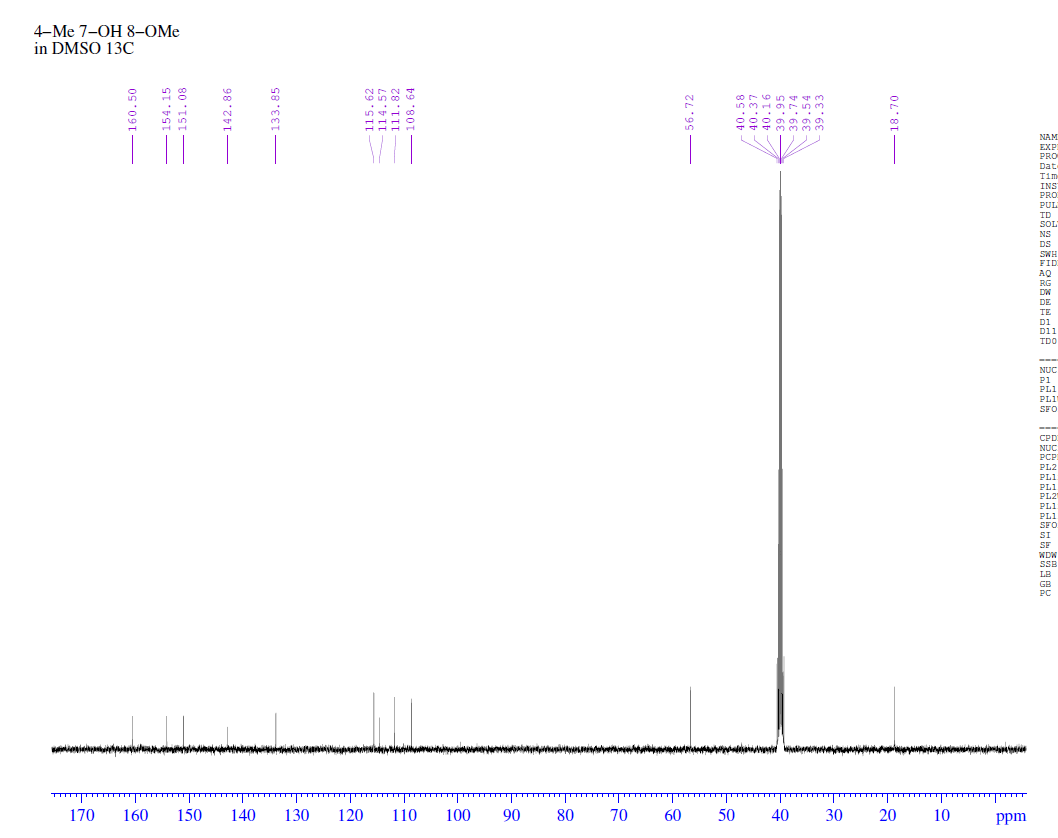


# Fig. S2 ^1^H NMR and ^13^C NMR spectra for 8-methoxy-4-MDPN

^1^H NMR (400 MHz, DMSO-*d*_6_)


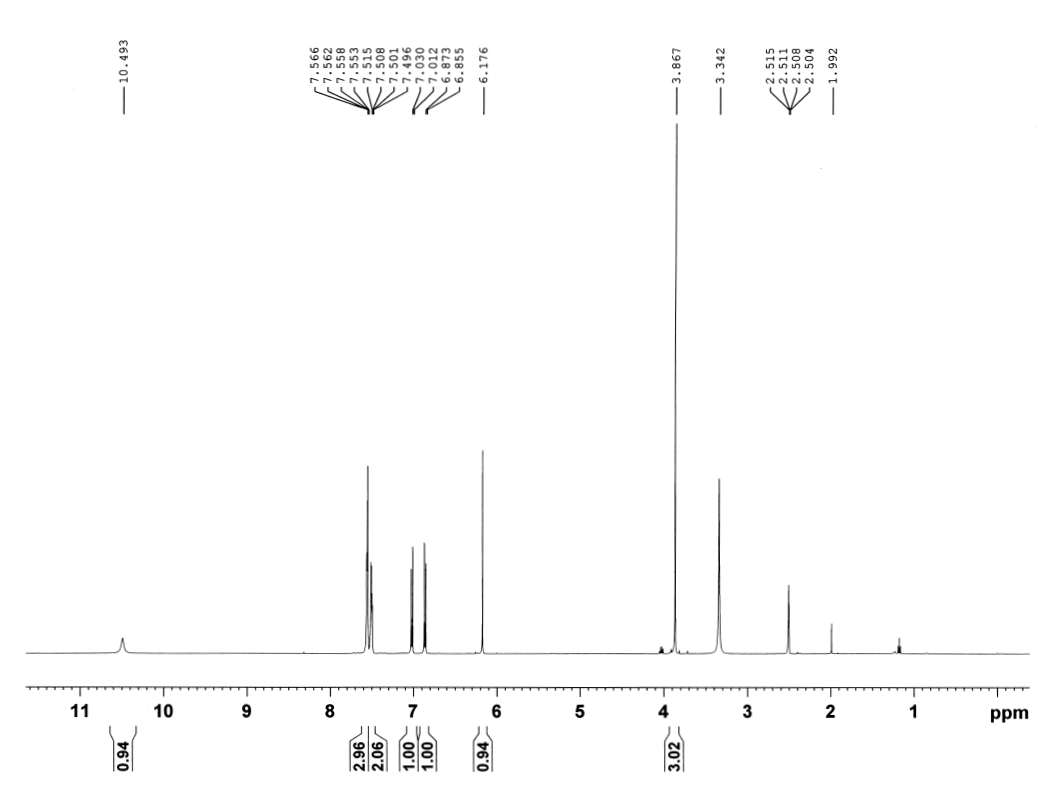


# ^13^C NMR (100 MHz, DMSO-*d*_6_)

#
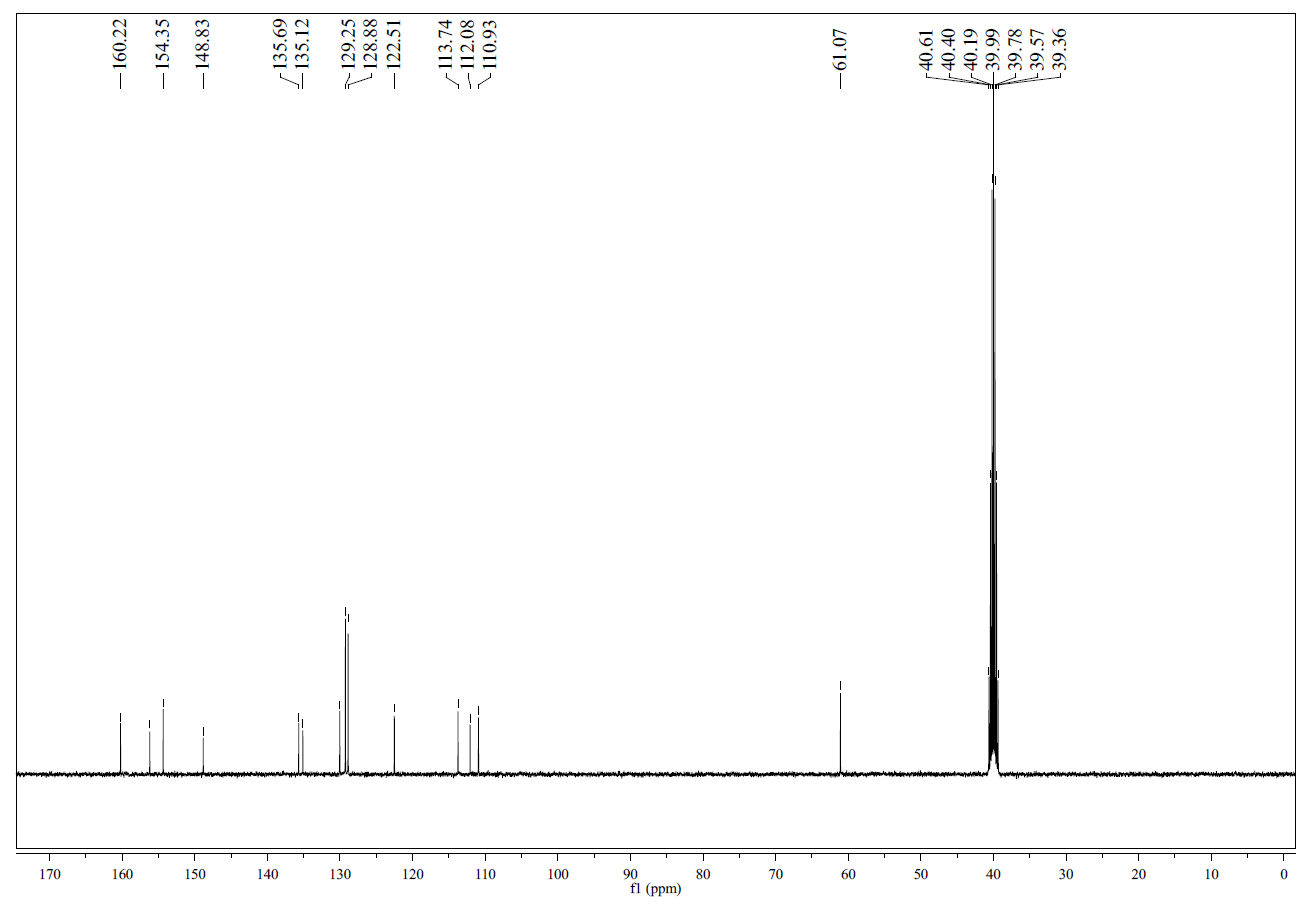


# Fig. S3 ^1^H NMR and ^13^C NMR spectra for 8-methoxy-4-PDPN

^1^H NMR (400 MHz, DMSO-*d*_6_)


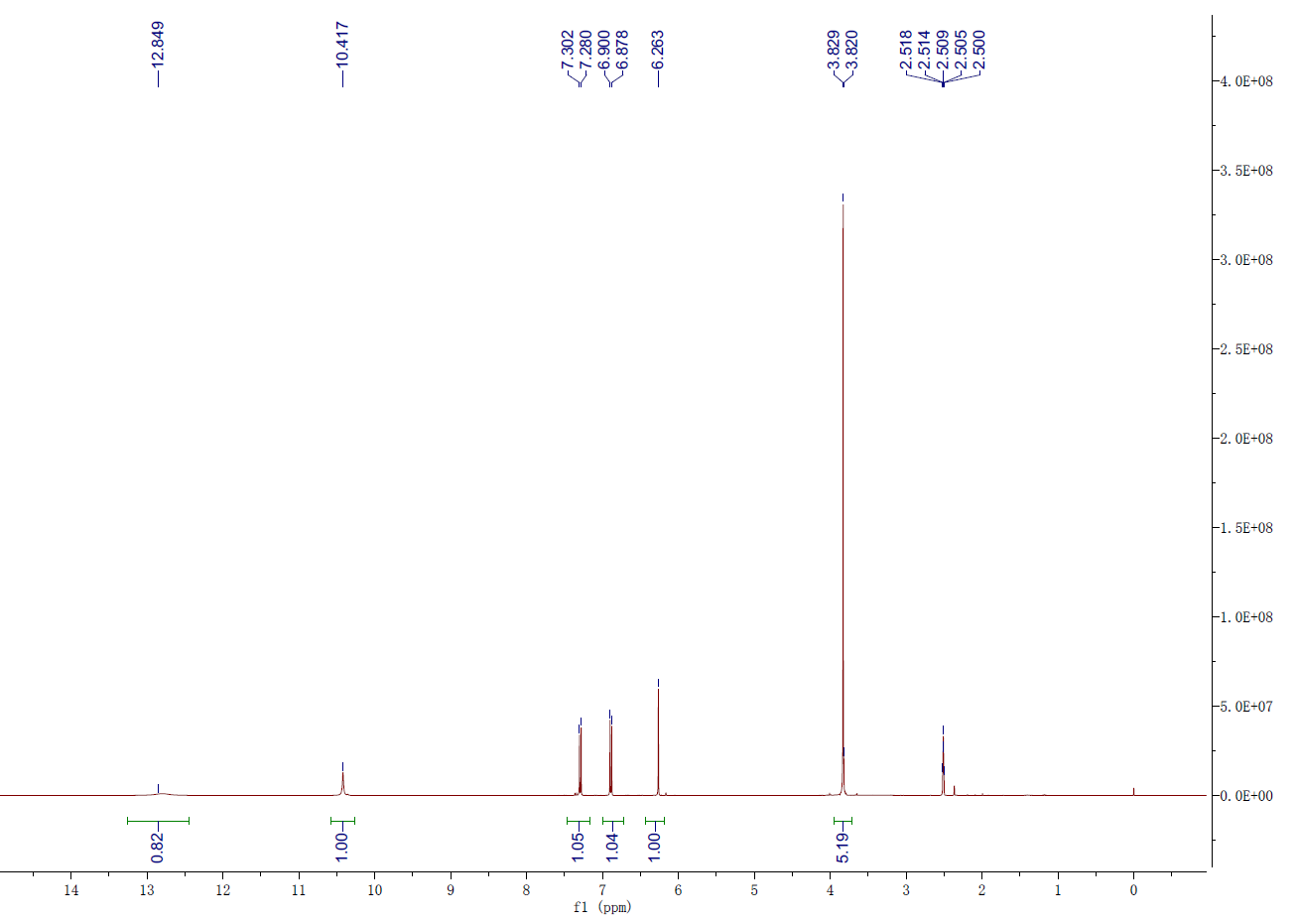


# ^13^C NMR (100 MHz, DMSO-*d*_6_)

#
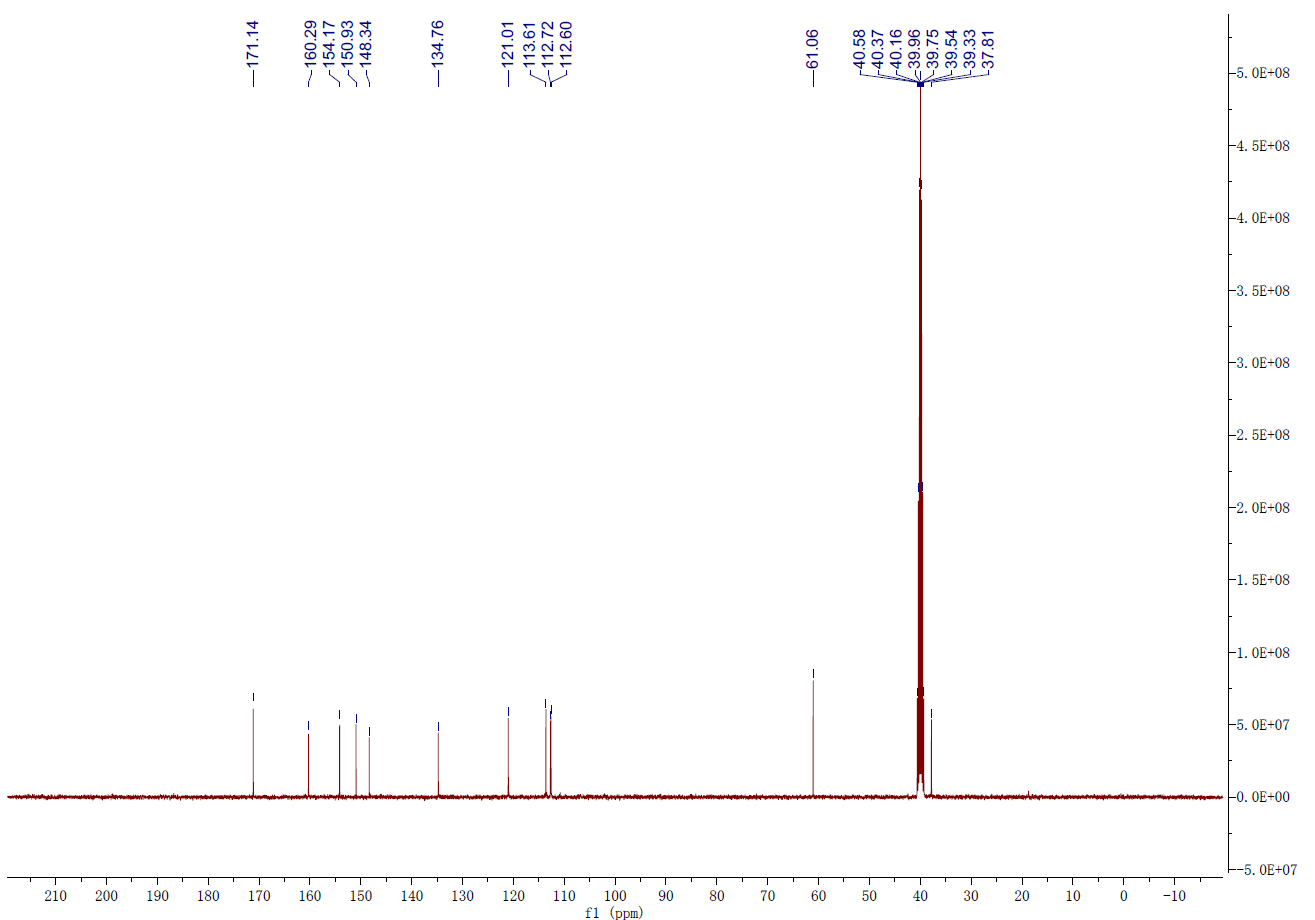


# Fig. S4 ^1^H NMR and ^13^C NMR spectra for 8-methoxy-4-ADPN

^1^H NMR (400 MHz, DMSO-*d*_6_)


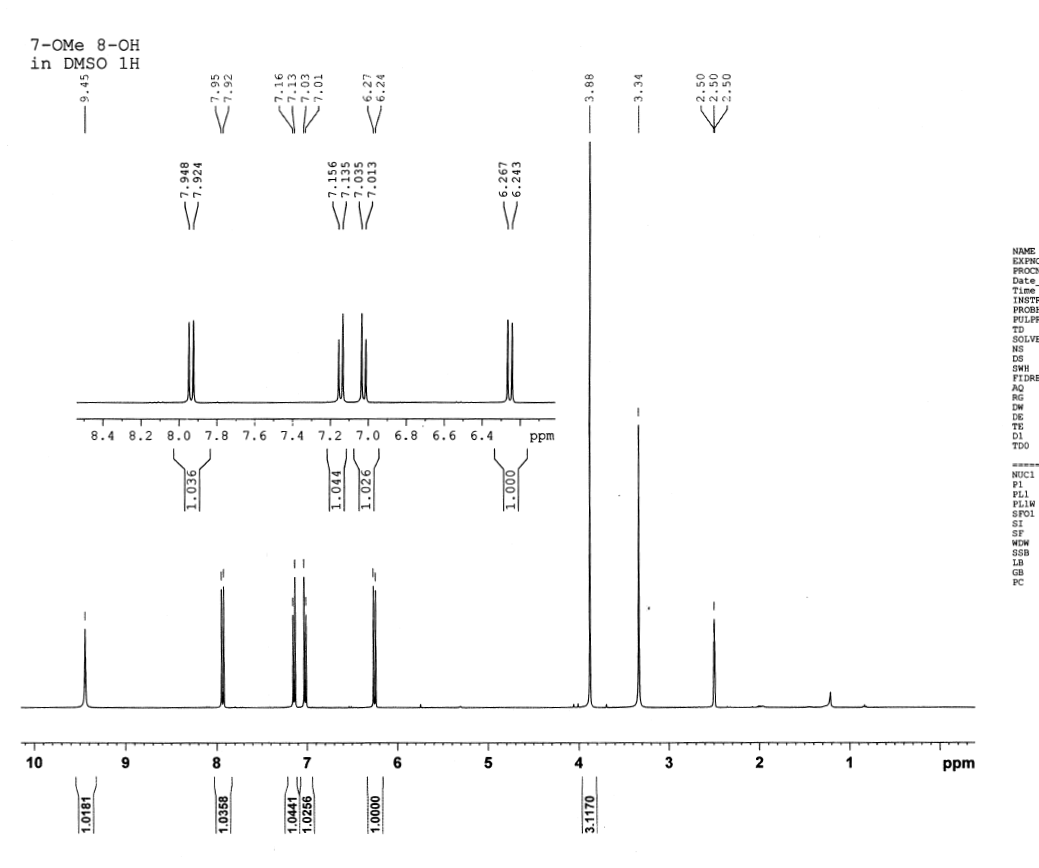


# ^13^C NMR (100 MHz, DMSO-*d*_6_)

#
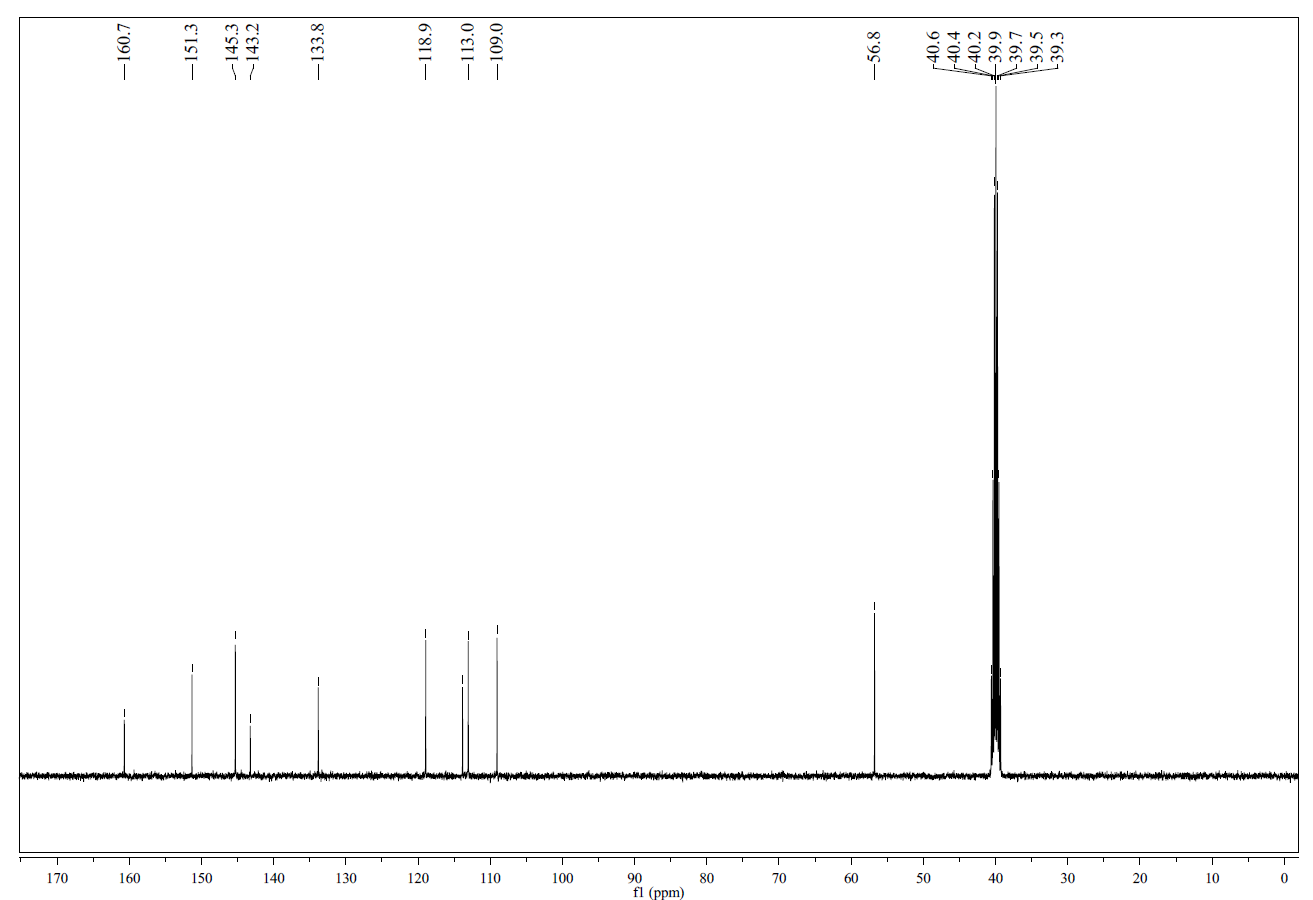


Fig. S5 ^1^H NMR and ^13^C NMR spectra for 7-methoxy daphnetin

^1^H NMR (400 MHz, DMSO-*d*_6_)


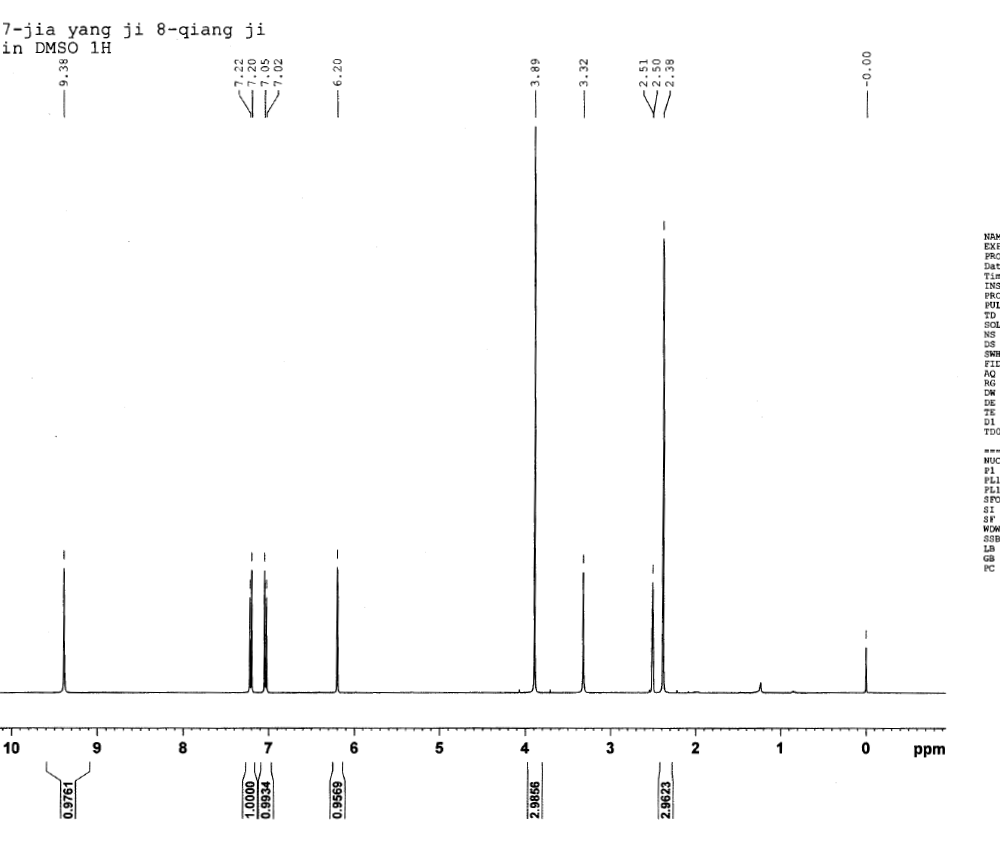


# ^13^C NMR (100 MHz, DMSO-*d*_6_)

#
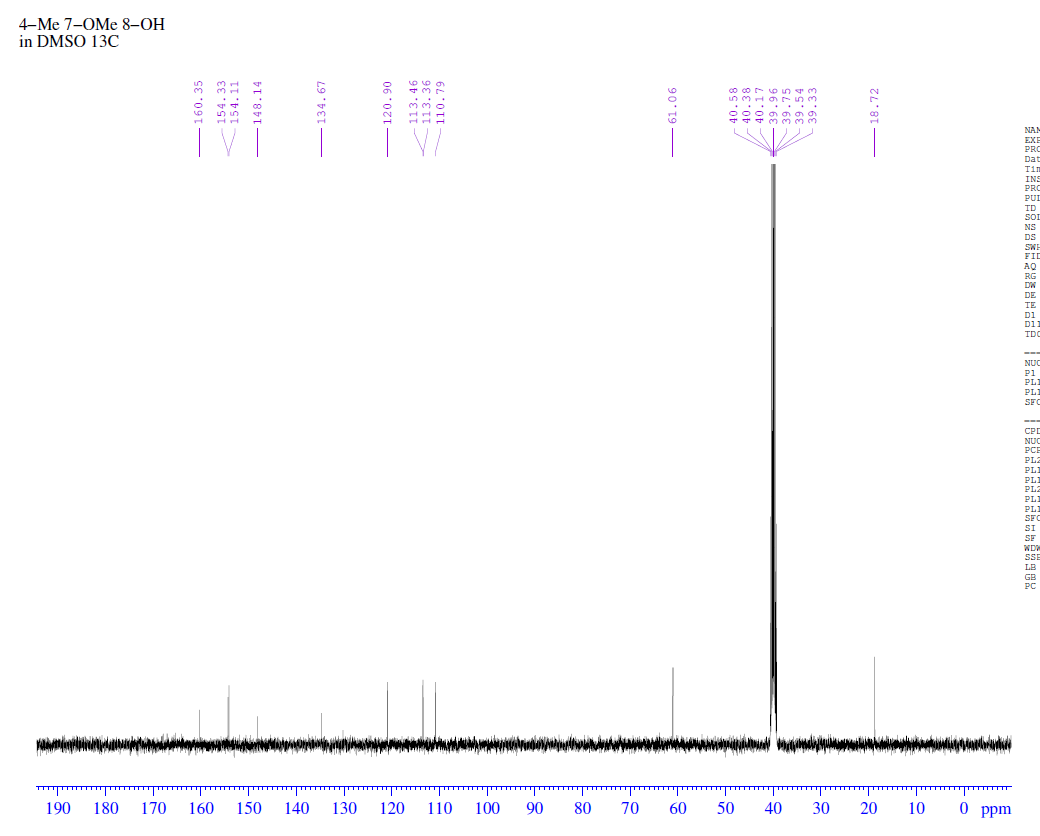


Fig. S6 ^1^H NMR and ^13^C NMR spectra for 7-methoxy-4-MDPN

^1^H NMR (400 MHz, DMSO-*d*_6_)


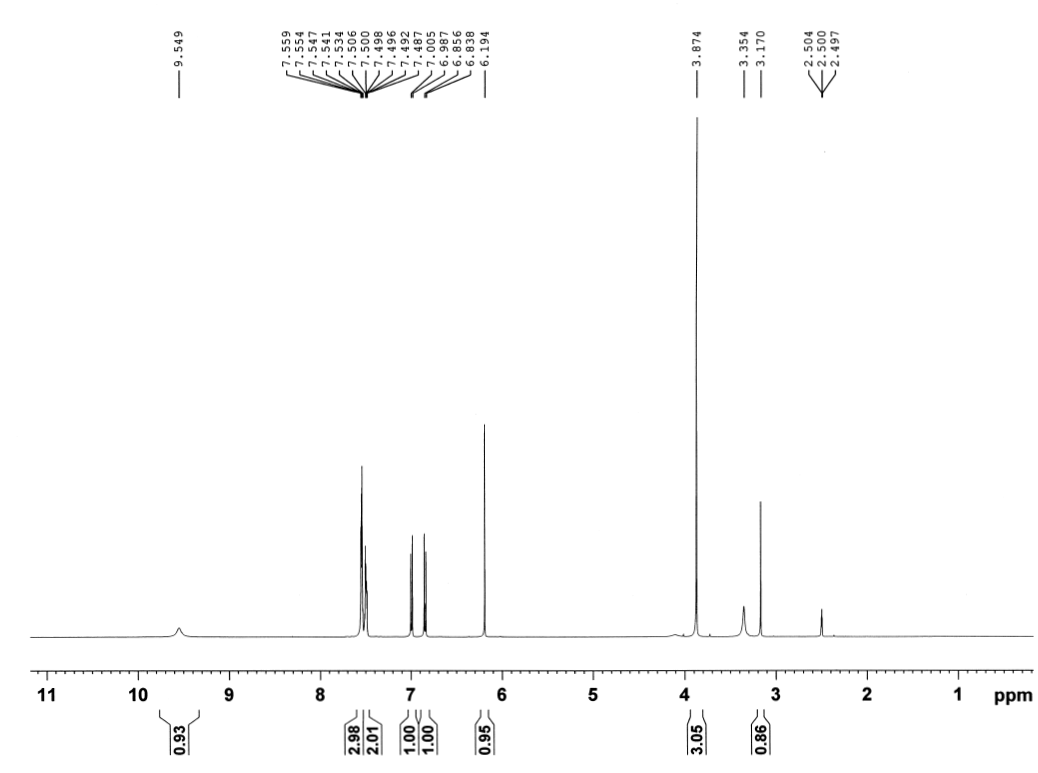


# ^13^C NMR (100 MHz, DMSO-*d*_6_)

#
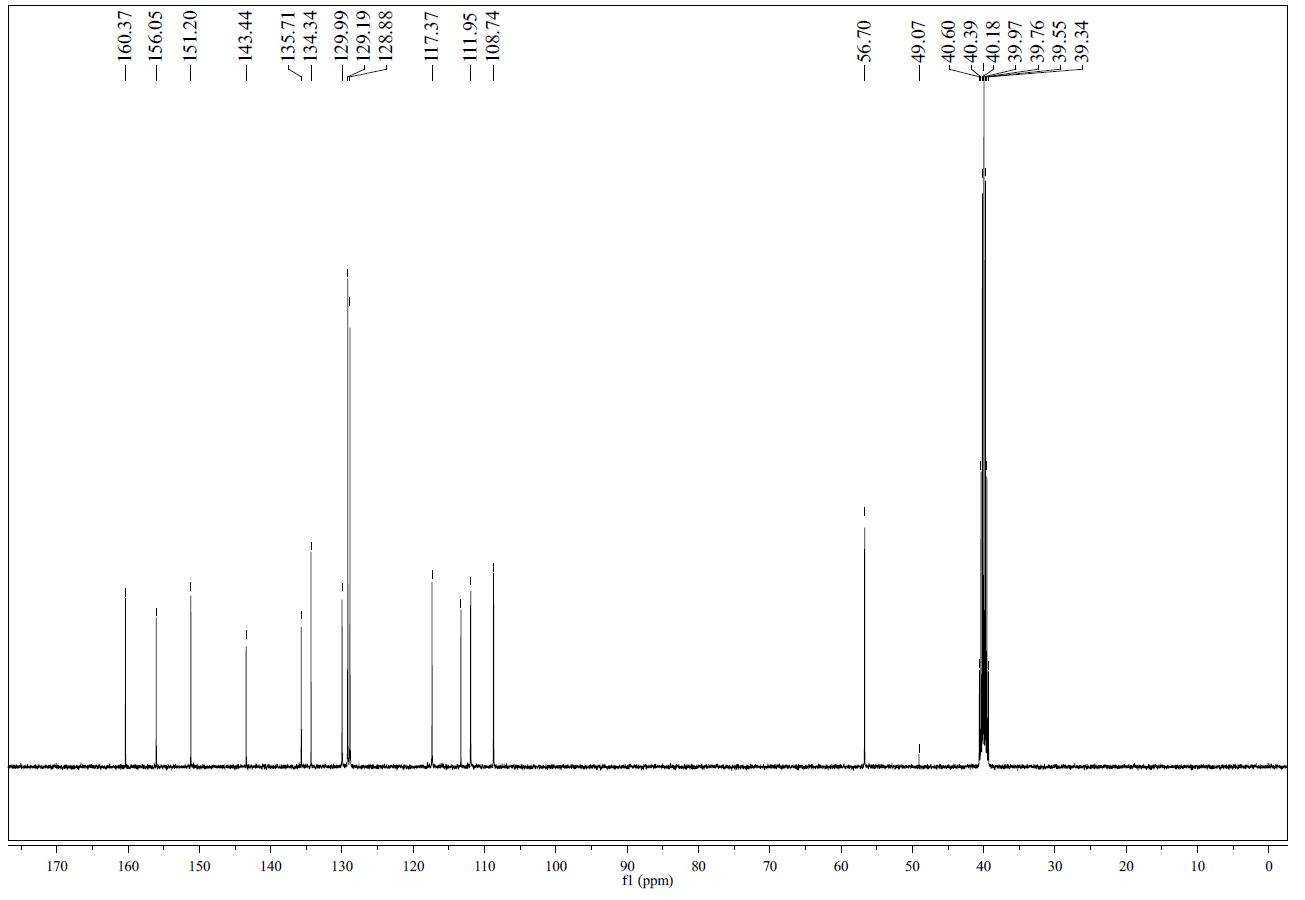


Fig. S7 ^1^H NMR and ^13^C NMR spectra for 7-methoxy-4-PDPN

^1^H NMR (400 MHz, DMSO-*d*_6_)


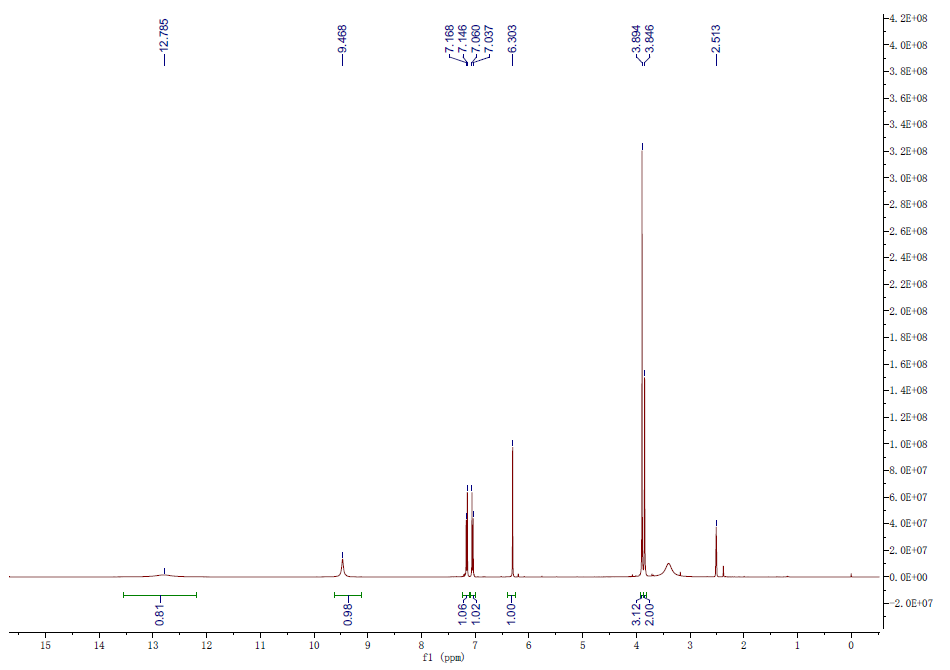


# ^13^C NMR (100 MHz, DMSO-*d*_6_)

#
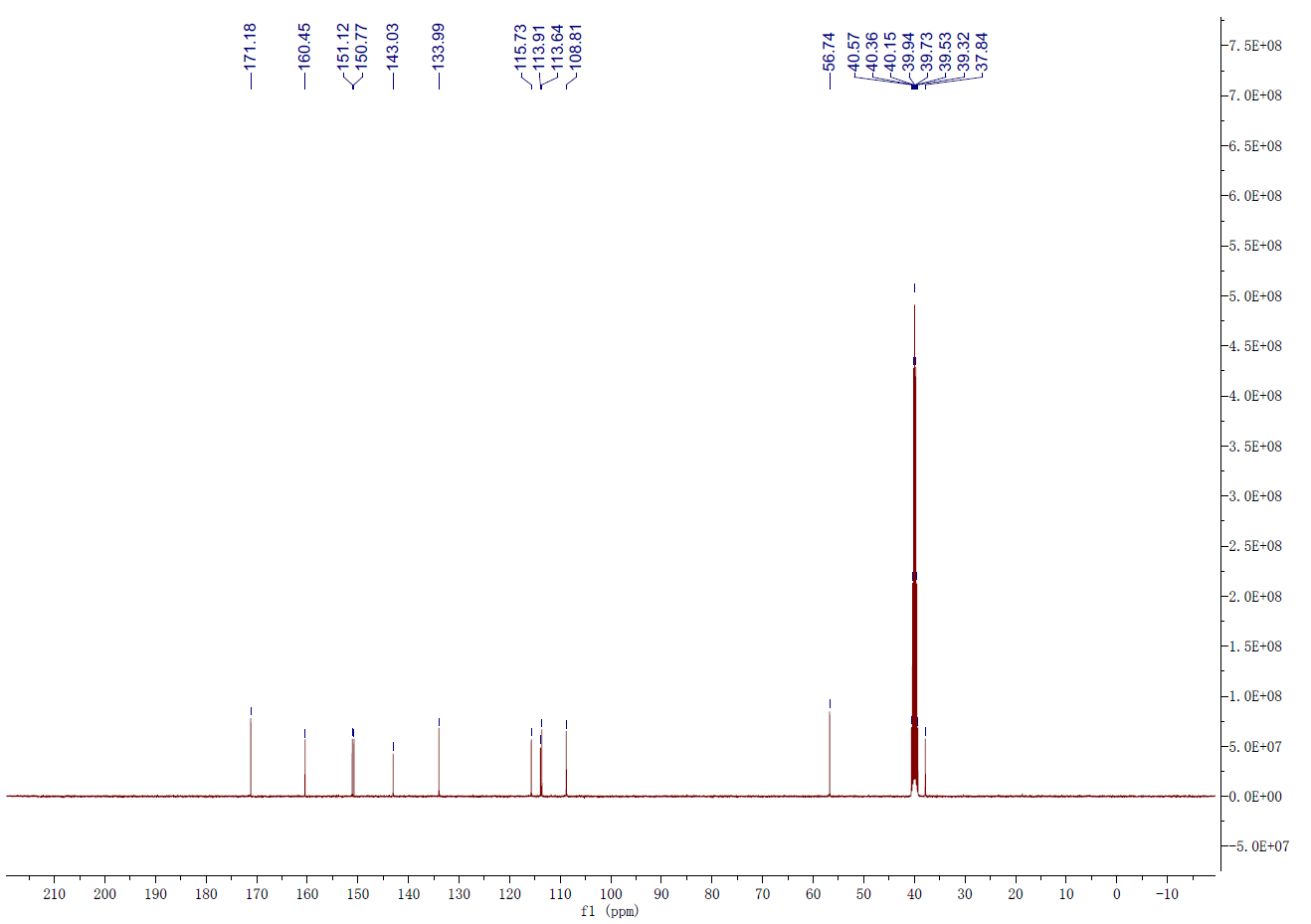


# Fig. S8 ^1^H NMR and ^13^C NMR spectra for 7-methoxy-4-ADPN
